# Multi-band network fusion for Alzheimer’s disease identification with functional MRI

**DOI:** 10.3389/fpsyt.2022.1070198

**Published:** 2022-12-15

**Authors:** Lingyun Guo, Yangyang Zhang, Qinghua Liu, Kaiyu Guo, Zhengxia Wang

**Affiliations:** School of Computer Science and Technology, Hainan University, Haikou, China

**Keywords:** functional brain networks, signal decomposition, network fusion, resting state fMRI, Alzheimer diagnosis

## Abstract

**Introduction:**

The analysis of functional brain networks (FBNs) has become a promising and powerful tool for auxiliary diagnosis of brain diseases, such as Alzheimer’s disease (AD) and its prodromal stage. Previous studies usually estimate FBNs using full band Blood Oxygen Level Dependent (BOLD) signal. However, a single band is not sufficient to capture the diagnostic and prognostic information contained in multiple frequency bands.

**Method:**

To address this issue, we propose a novel multi-band network fusion framework (MBNF) to combine the various information (e.g., the diversification of structural features) of multi-band FBNs. We first decompose the BOLD signal adaptively into two frequency bands named high-frequency band and low-frequency band by the ensemble empirical mode decomposition (EEMD). Then the similarity network fusion (SNF) is performed to blend two networks constructed by two frequency bands together into a multi-band fusion network. In addition, we extract the features of the fused network towards a better classification performance.

**Result:**

To verify the validity of the scheme, we conduct our MBNF method on the public ADNI database for identifying subjects with AD/MCI from normal controls.

**Discussion:**

Experimental results demonstrate that the proposed scheme extracts rich multi-band network features and biomarker information, and also achieves better classification accuracy.

## 1 Introduction

Alzheimer’s disease (AD) is an irreversible neurodegenerative disease that severely impacts the quality of life for patients (1). As a non-invasive measure for detecting brain abnormalities, functional brain network (FBN), derived from resting state magnetic resonance imaging (rs-fMRI), provides a valuable opportunity for early intervention and control of AD disease. Previous studies usually divide the brain of patients into several regions of interest (ROI) through a certain brain template. Then, the FBN is constructed by calculating the full band Blood Oxygen Level Dependent (BOLD) signals correlation coefficients among these ROIs. However, a single band is not sufficient to capture the diagnostic and prognostic information contained in multiple frequency bands.

In practice, BOLD signals based on different frequencies have different physiological significance. As early as 1995, researchers found that there is a correlation between low-frequency BOLD signals in certain brain regions (2). In 2011, Baria et al. divided the BOLD signal into four frequency bands to study the energy of each band and its distribution in the whole brain. They found that the signals in the 0.01–0.05 Hz frequency band are mainly distributed in the prefrontal, parietal, and occipital cortices; the signals in the 0.05–0.1 Hz frequency band are mainly distributed in the thalamus and basal ganglia; the signals in the 0.1–0.15 Hz frequency band are mainly distributed in the insula and temporal cortex; the signals in the 0.15–0.2 Hz frequency band are also distributed in the insula and temporal cortex (3). Most studies focused on the BOLD signal at (0.01–0.08) Hz, a range in which frequencies vary between brain regions.

In addition to the different physiological significance, many studies found that the use of frequency division in estimating FBNs with different frequency bands can achieve a variety of descriptions of FBN structures. For example, Zhang et al. calculate the node statistics (e.g., node degree, node path length, and betweenness centrality) of FBNs estimated by different bands and discover that the structural characteristics of different frequency bands are significantly different (4). Song et al. decomposed the time series of each voxel and found that ReHo in cortical areas was higher and more frequency-dependent than those in the subcortical regions (5). Li et al. found that compared with the healthy control group, the functional connectivity of patients with temporal lobe epilepsy in δ, θ, low α, and β bands was significantly increased, and the value of the weighted small-world measure in θ band was significantly decreased (6). Besides, studies have found that different band-based FBNs used for disease diagnosis achieved different classification results (7). The explanation is that FBNs based on different frequencies have different discrimination abilities.

Since the different information brought by different frequency bands, it is a good perspective to decompose the BOLD signal into multiple bands for constructing multiple FBNs and fuse the features of every FBN. For example, Zou et al. extract the temporal, spatial, and spatial-temporal variability features of functional networks in each frequency band and fused them into a set of feature vectors for schizophrenia classification (8). Zuo et al. proposed a deep multi-fusion framework with classifier-based feature synthesis to automatically fuse multi-modal medical images. They validated the approach for brain disease classification using the fused images and illustrated that the improvement in classification performance is due to the adoption of the fusion strategy (9). However, these feature fusion methods have limited interpretability, which does not provide a good biomarker for the diagnosis of brain diseases. More important, both global-and local-level features extracted from FBNs tend to capture different network properties, which requires prior knowledge and thus makes the feature design an intractable problem.

Different from feature fusion, network fusion can obtain the diverse information of multiband-based FBNs and eliminate the redundant information caused by the correlation between different feature sets. Considering the varied characteristics of FBNs in different frequency bands, we propose a novel multi-band network fusion framework (MBNF) to estimate information-rich multi-frequency FBNs. Specifically, our framework can be summarized in the following steps: (1) using ensemble empirical mode decomposition (EEMD) to decomposed the bold signal into high and low-frequency bands adaptively; (2) fusing FBNs constructed by the two frequency bands into a multi-band fusion network by similarity network fusion (SNF); (3) extracting the features of the fused networks and employing the Support Vector Machines (SVM) for classification.

The rest of the paper is organized as follows. In Section “2 Material and methods,” we present the experimental data and the proposed method. In Section “3 Experiment,” we design the experiment and compare it with other methods. In Section “4 Discussion,” we discuss the effect of different signal decomposition methods, and different fusion methods on the classification results. Then, we propose the limitations of the work and future research directions. In Section “5 Conclusion,” we conclude this article.

## 2 Materials and methods

In this section, we first introduce data acquisition and preprocessing in detail. Then the overall process of brain disease classification based on the MBNF framework is presented in the following parts.

### 2.1 Dataset description and image preprocessing

In this paper, we evaluate our proposed scheme based on the dataset from the Alzheimer’s Disease Neuroimaging Initiative (ADNI), which divides MCI into two subcategories, early MCI (eMCI) and late MCI (lMCI), and previous studies have shown that lMCI has a high potential for transition to AD. The datasets contain 154 normal controls (NCs), 165 eMCI, 145 lMCI, and 99 AD. The scan parameters of these data as listed below: in-plane image resolution is 2.29–3.31 mm and the thickness of each slice is 3.31 mm. The Echo time (TE) of the slice is 30 ms, and the repetition time (TR) is 2.2–3.1 s. Each subject’s scan consisted of 140 volumes. The detailed demographic information is shown in Table 1.

**TABLE 1:** Demographic information of the involved 563 rs-fMRI subjects from the Alzheimer’s Disease Neuroimaging Initiative (ADNI) database.

Category	Scan #	Age (Years)	Gender (M/F)
AD	99	75.04± 7.71	55/44
eMCI	165	72.03± 7.26	73/92
lMCI	145	71.99± 7.67	95/50
NC	154	75.36± 6.16	67/87

The values are denoted as mean ± standard deviation. M/F: male/female.

We used FSL FEAT software which is a standard pipeline to process the rs-fMRI scans (10). We first cast aside the first 3 volumes to allow signal stabilization. For the remaining 137 volumes, we corrected the slice time and motion to avoid interference with the data and eliminate the impact of head motion. Then, we striped the structural skull according to the T1-weighted MRI. We use the processed image to align with the Montreal Neurological Institute (MNI) space. All subjects are processed with band-pass filtering at frequency intervals of [0.015, 0.15 Hz]. And then we regress the nuisance signals which contain motion parameters, white matter, and cerebrospinal fluid. Furthermore, a Gaussian kernel with full-width-at half-maximum (FWHM) of 6 mm is used to smooth the data. It is worth noting that we did not perform scrubbing to data because this would introduce additional artifacts. At last, the brain space of fMRI scans is partitioned into 116 pre-defined ROIs using the Automated Anatomical Labeling (AAL) template (11). For each subject, the bold signals are extracted from each ROI, and then normalized as following:


(1) $$\text{r}\,\left(x\right)=\frac{\left(x-\mu_{i}\right)}{\sigma_{i}}$$

where *x* denotes the time point signal from the *i*-th ROI. μ_*i*_ represents the mean of the *x* and σ_*i*_ denote the standard deviation of the *x*.

### 2.2 The multi-band network fusion framework

**FIGURE 1:**
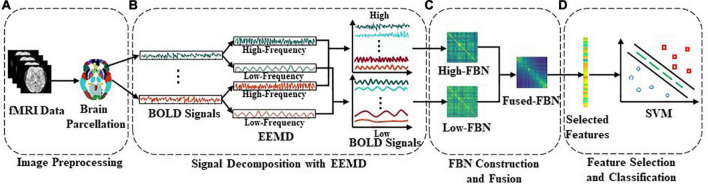
Flowchart of the proposed multi-band network fusion framework (MBNF) scheme for brain disease classification, including four major parts: **(A)** data acquisition and image pre-processing; **(B)** blood oxygen level dependent (BOLD) signal decomposition based on ensemble empirical mode decomposition (EEMD); **(C)** functional brain networks (FBN) construction and fusion; and **(D)** feature selection and classification.

In this section, we introduce the multi-band network fusion framework (MBNF) scheme for brain disease diagnosis. As shown in Figure 1, the MBNF contains three major parts: (1) BOLD signal decomposition based on EEMD; (2) FBN construction and fusion; and (3) feature selection and classification.

#### 2.2.1 BOLD signal decomposition based on EEMD

Previous studies typically used band-pass filters (e.g., wavelet transform) to acquire multi-band signals. However, since the frequency characteristics of the BOLD signal are complex, traditional band-pass filters are unsuitable. Therefore, Huang et al. propose a novel adaptive signal time-frequency processing method called empirical mode decomposition (EMD) (12). Different from wavelet transform which needs to set the feasible decomposition layers in advance, EMD can decompose signals adaptively according to the time characteristics of data. Specifically, EMD can decompose the non-stationary time series into a group of Intrinsic Mode Functions (IMF) components, which are oscillatory functions with time-varying frequencies and can reflect the local characteristics of non-stationary signals (13).

**FIGURE 2:**
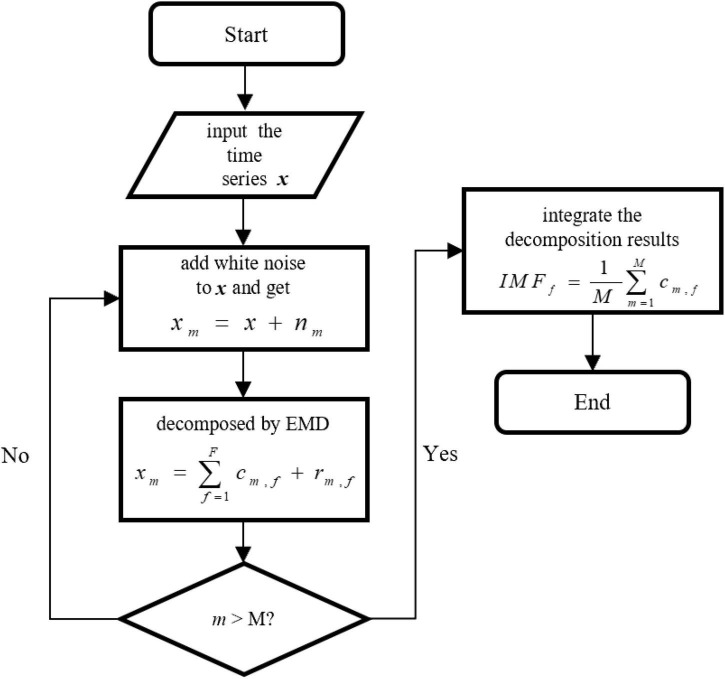
The algorithm flowchart of ensemble empirical mode decomposition (EEMD).

In practice, the mode aliasing problem can occur during the execution of EMD, which leads to mistakes for subsequent feature extraction, model training, and pattern recognition. To solve this problem, the ensemble empirical mode decomposition (EEMD), an improved method of EMD, is performed for signal decomposition in the proposed MBNF method (14). Specifically, EEMD adds different white noises with the same amplitude to alter the extreme point characteristics of signals (15). Figure 2 presents the algorithm flowchart. In Figure 2, x is the original signal, *n*_*m*_ represents the *m*−*th* additive white noise sequence, *c*_*m*,*f*_ represents the *f*−*th* IMF component obtained by decomposition after adding white noise for the *m*−*th* time, *f* is the number of IMF components, *r*_*m*,*f*_ is the residual function, and *M* is the average number of corresponding IMF components after multiple decomposition.

**FIGURE 3:**
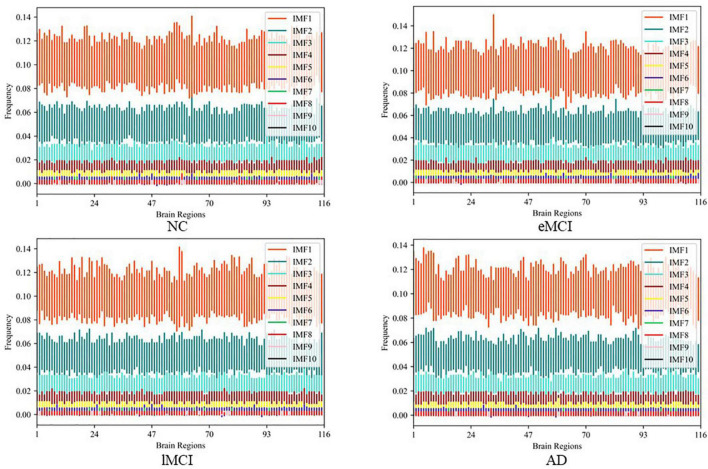
The average frequency of intrinsic mode functions (IMF) components in every brain region in different categories.

After obtaining the IMF components, we transformed the IMF time-domain signals of each brain region into frequency-domain signals to display the frequency-domain range of each IMF component. Note, since EEMD decomposition is adaptive, the number of IMF components after signal decomposition in each brain region may be different. Specifically, we calculate the average frequency of IMF components in every brain region and show the total results of all subjects in different categories (i.e., eMCI, lMCI, AD, and NC) in Figure 3. We can observe in Figure 3 that no matter in which category, the IMF1 component is about 0.06–0.16 Hz, while the average frequency of other IMF components is less than 0.1 Hz. In order to facilitate the construction of a FBN for subsequent analysis, IMF1 components are used as high-frequency BOLD signals, and the remaining IMFs components are integrated together as low-frequency BOLD signals.

#### 2.2.2 FBN construction and fusion

Once we obtain the high/low-frequency BOLD signals of each ROI, we utilize the two types of signals to estimate different FBNs, which provides an effective tool to compare different subjects and to mine biomarkers of neurological/mental disorders. Note, we perform different methods to construct FBNs in the follow-up experiment for verifying the robustness of our method. In recent decades, a number of methods have been developed for constructing FBNs, among which the representative is Pearson’s correlation (PC) and sparse representation (SR) (16–18).

Specifically, denote *P* (*P* = 116 in this work) is the number of ROIs and *T* (*T* = 137 in this work) is the total number of temporal image volumes. For any *i*,*j* (*i*,*j* = 1,⋯,*P*), *W*_*ij*_ is the functional connectivity between a pair of ROIs *i* and *j*. The calculation formula of PC-based functional connectivity is as follows:


(2) $$W_{i\,j}^{P\,C}=\frac{{\left(x_{i}-{\bar{x}}_{i}\right)}^{T}\,\left(x_{j}-{\bar{x}}_{j}\right)}{\sqrt{{\left(x_{i}-{\bar{x}}_{i}\right)}^{T}\,\left(x_{i}-{\bar{x}}_{i}\right)}\,\sqrt{{\left(x_{j}-{\bar{x}}_{j}\right)}^{T}\,\left(x_{j}-{\bar{x}}_{j}\right)}}$$

where *x*_*i*_ ∈ *R^T^* represents the time series of the *i*th ROI, ${\bar{x}}_{i}\in R^{T}$ is the corresponding mean vector of *x*_*i*_ . Another FBN construction method is SR, which is an *l*_1_ -regularized linear regression. The mathematical model can be obtained by the following objective function:


(3) $$\min_{W^{S\,R}}\sum_{i=1}^{P}\left({||x_{i}-\sum_{j\neq i}W_{i\,j}^{S\,R}\,x_{j}||}^{2}+\lambda \,\sum_{j\neq i}\left|W_{i\,j}^{S\,R}\right|\right)$$

where λ is a regularized parameter. Note, the same methods are performed for high-frequency FBN and low-frequency FBN.

After FBN construction, we perform the similarity network fusion (SNF) method to fuse high/low-frequency FBNs for obtaining complementary information of multi-frequency bands. The similarity fusion network is robust to noise and can obtain useful information from fewer samples (19, 20). For high-frequency FBN (*W^High^*) and low-frequency FBN (*W^Low^*), we construct similarity matrix *S^High^* and *S^Low^* separately. Note, similarity matrix is a sparse kernel matrix encoding its own sparse strong connections. For every similarity matrix *S*, we use the K-nearest neighbors (KNN) to measure the local affinity, and set the similarity between non-adjacent points to zero. The calculation formula of similarity value between a pair of ROIs *i* and *j* is as follows:


(4) $$S_{i\,j}=\left\{ \begin{array}{cc} W_{i\,j}, & i\,f\,i\in K\,N\,N_{j}\\ 0, & o\,t\,h\,e\,r\,w\,i\,s\,e \end{array}\right.$$

where *KNN*_*j*_ represents a set of K-nearest neighbors of the ROI *j* in *W*. Similar to previous study (21), we set the number of nearest neighbors to 11.

Based on the sparse kernel matrixes *S^High^* and *S^Low^*, we fuse them into a single network using nonlinear methods. Each similar network needs to be updated iteratively to make it more similar to another network. For example, *S^High^* could be iteratively updated as follows:


(5) $${\left(W^{H\,i\,g\,h}\right)}^{g+1}=S^{H\,i\,g\,h}\times {\left(W^{L\,o\,w}\right)}^{\left(g\right)}\times {\left(S^{H\,i\,g\,h}\right)}^{T}$$

where *g* is the number of iterations, (*W^Low^*)^(*g*)^ represents the *W^Low^* after *g*th iteration.

Because different networks carry distinct frequency information, *W^High^* can integrate the information provided by *W^Low^* after several iterative learnings. At the same time, the sparse kernel matrix *S* guides the iterative process through the strongest connections of *W*, and thus can reduce the noise effectively. Iteration stops when the converged network is close to stopping changing. Because different networks carried different frequency information, fusion networks integrated the information provided by different frequency networks. When the iterative fusion network was almost constant, the network stopped iterating. Specifically, the fusion network stops updating in the process of iteration when it satisfies the formula (6):


(6) $$||{\left(W^{H\,i\,g\,h}\right)}^{g+1}-{\left(W^{H\,i\,g\,h}\right)}^{g}||\leq 0.01$$

Finally, we obtain the fusion network by averaging two networks. The fusion network is as follows:


(7) $$W^{F\,u\,s\,e\,d}=\frac{{\left(W^{H\,i\,g\,h}\right)}^{\prime }+{\left(W^{L\,o\,w}\right)}^{\prime }}{2}$$

where (⋅)′ represents the last updated matrix.

#### 2.2.3 Feature selection and classification

**FIGURE 4:**
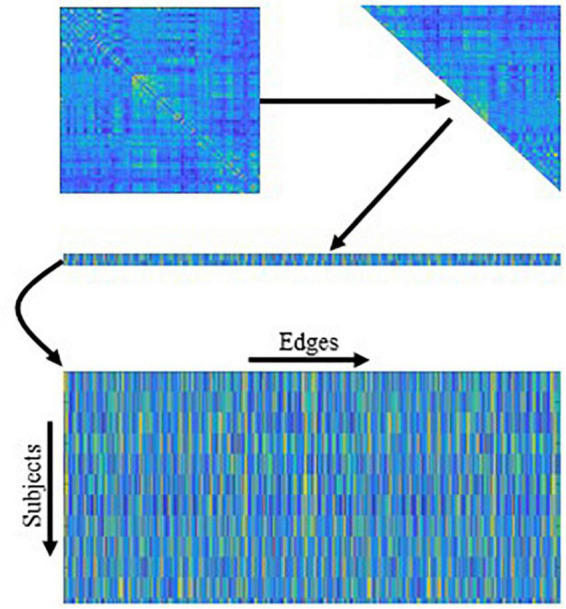
The mechanism of traditional edge feature extraction in functional brain network (FBN). The network adjacency matrix from each subject is first mapped onto a vector by removing the redundant part if the matrix is symmetric, and then the vectors from all subjects are rearranged together as an input of the following feature selection methods.

Once we obtain the fused FBNs for all subjects, the subsequent task is to extract/select the most discriminative features according to the FBNs for disease classification. Currently, there are two categories of features based on different granularities in FBN analysis, including node-level and edge-level features. Since the node-level features tend to capture different network properties that caused the extra prior knowledge to design effective features, we use the edge-level feature (i.e., functional connectivity between ROIs) in our experiment. As shown in Figure 4, we concatenate the upper triangle of the obtained fused FBNs into an edge vector (removing the redundant part if the adjacent matrix is symmetric), and then pile up the edge vectors from all subjects into a feature matrix for subsequent classification tasks. Besides, in order to remove redundant information in these features, *t*-test is used for feature selection (*P* < 0.05).

Finally, considering that small changes in different steps (FBN construction, feature selection, and classification) will have an impact on the end results, it is difficult to conclude which step contributes further to the final accuracy. Therefore, the simplest and most popular classifier support vector machine (*C* = 1) is performed to classify the AD/MCI from NC. In addition, the reason for using SVM instead of deep learning is the latter often requires very large data sets. It is challenging to train a good model and tune the hyper-parameters when there are not enough training samples (subjects).

## 3 Experiment

In this section, we first introduce the competing methods with our proposed scheme and the settings of our experiment. Then the experiment result is analyzed in detail.

### 3.1 Competing methods

In the experiments, we compare our proposed MBNF with several schemes, including (1) Full-Band, a scheme based on FBN construction by a full BOLD signal; (2) Low-Band, a scheme based on FBN construction by the low band after BOLD signal decomposition; (3) High-Band, a scheme based on FBN construction by the high band after BOLD signal decomposition; (4) MBNF, our proposed scheme. For a fair comparison, we employ *t*-test (*p* < 0.05) to select discriminative features and then use SVM (*C* = 1) for brain disease classification for all competing schemes. Besides, two FBN construction methods mentioned in 2.2.2 are performed in our experiment to further indicate the effectiveness of our method.

### 3.2 Experimental settings

We designed four classification tasks to evaluate the performance of our method and four competing schemes, which are as follows: (1) eMCI vs. NC (2) lMCI vs. NC (3) AD vs. NC (4) eMCI vs. lMCI. Then, three evaluation metrics are employed for evaluating the classification performance of all methods, including classification accuracy (ACC), sensitivity (SEN), and specificity (SPE), which are defined as follows:


(8) $$A\,C\,C=\frac{T\,P+T\,N}{T\,P+F\,P+T\,N+F\,N}\times 100\%$$

(9) $$S\,E\,N=\frac{T\,P}{T\,P+F\,N}\times 100\%$$

(10) $$S\,P\,E=\frac{T\,N}{T\,N+F\,P}\times 100\%$$

where TP, TN, FP, and FN represent true positive, true negative, false positive, and false negative, respectively. In addition to the above, we also add the area under the receiver operating characteristic curve (AUC) as another metric.

In our experiment, a 5-fold cross-validation (CV) is adopted to evaluate the generalization capability of the different methods. Besides, considering the hyper-parameters (i.e., sparsity) involved in the FBN construction methods may significantly affect the ultimate classification results, we select optimal parametric values by a grid search in a large range. For the regularized parameter λ in SR, we use 20 candidate values in [0.1, 0.15, 0.2, …, 0.95, 1]. Although PC is parameter-free. For a fair comparison, we perform a thresholding parameter in PC by preserving a percentage of connectivity with strongest correlation. To be consistent with other methods, we set up 20 sparsity from a candidate set [5%, 10%, …95%, 99%]. For example, 100% means all edges are preserved, and 90% means 10% weak edges are removed. Then, an inner-5-fold CV on the training data to determine the optimal sparsity, which is based on the classification accuracy in each inner loop. For fairness, we also employed inner-5-fold CV strategy in other competitive methods compared with MBNF. Note, we perform the 5-fold CV process 1,000 times independently to avoid random errors introduced in cross-validation, and the mean and standard deviation of the classification results are reported in Table 2. To illustrate the statistical significance of the results, we perform a paired *t*-test (*p* < 0.05) on the results of the methods involved and then use “*” to mark the results better than the other methods.

**TABLE 2:** Classification performance of four schemes in four classification tasks based on Pearson’s correlation (PC) construction method (mean ± standard deviation).

Task	Scheme	ACC (%)	SEN (%)	SPE (%)	AUC (%)
eMCI vs. NC	Full-band	84.29 ± 1.94	88.60 ± 1.47	79.92 ± 1.52	90.94 ± 0.47
	Low-band	78.98 ± 1.04	81.82 ± 1.07	76.94 ± 2.31	87.86 ± 0.96
	High-band	83.94 ± 1.28	88.61 ± 1.31	78.24 ± 1.17	89.58 ± 0.83
	MBNF	90.60 ± 1.56*	92.72 ± 1.95*	88.48 ± 2.14*	97.50 ± 0.25*
lMCI vs. NC	Full-band	86.28 ± 2.02	87.95 ± 2.13	83.40 ± 1.22	82.43 ± 0.32
	Low-band	78.25 ± 1.18	80.48 ± 1.72	74.89 ± 1.83	87.03 ± 0.61
	High-band	84.61 ± 1.58	85.95 ± 1.52	80.03 ± 1.64	92.02 ± 1.05
	MBNF	91.98 ± 1.66*	93.34 ± 2.29*	90.23 ± 1.47*	97.12 ± 0.71*
eMCI vs. lMCI	Full-band	81.93 ± 2.11	**91.78 ± 1.81**	73.41 ± 2.23	88.19 ± 0.57
	Low-band	76.77 ± 0.98	74.67 ± 1.32	80.06 ± 1.37	82.43 ± 1.04
	High-band	75.80 ± 1.74	68.24 ± 2.01	83.32 ± 0.95	87.05 ± 1.11
	MBNF	90.64 ± 1.44*	86.02 ± 1.92	94.97 ± 2.02*	96.98 ± 0.46*
AD vs. NC	Full-band	90.49 ± 2.01	**87.95 ± 1.23**	83.40 ± 1.78	82.43 ± 0.41
	Low-band	80.63 ± 0.94	79.03 ± 1.44	90.97 ± 0.89	86.79 ± 0.52
	High-band	90.89 ± 1.27	81.38 ± 1.56	95.39 ± 2.01	96.90 ± 0.79
	MBNF	93.08 ± 1.85*	86.96 ± 1.35	96.73 ± 1.32*	98.58 ± 0.74*

*Denotes that the result of MBNF is significantly better than other competing schemes. Bold values indicate the best results in each task.

### 3.3 Classification results and analysis

Tables 2, 3 provide the classification results of four schemes in four tasks based on two FBN construction methods, and also shows some intriguing findings.

**TABLE 3:** Classification performance of four schemes in four classification tasks based on sparse representation (SR) construction method (mean ± standard deviation).

Task	Scheme	ACC (%)	SEN (%)	SPE (%)	AUC (%)
eMCI vs. NC	Full-band	84.31 ± 2.31	86.30 ± 1.85	81.84 ± 1.39	93.24 ± 0.56
	Low-band	77.43 ± 1.80	79.69 ± 1.36	76.01 ± 1.35	83.02 ± 0.68
	High-band	82.14 ± 1.53	85.85 ± 1.09	78.17 ± 1.91	90.76 ± 0.51
	MBNF	89.91 ± 1.75*	93.51 ± 1.75*	88.59 ± 1.84*	96.97 ± 0.41*
lMCI vs. NC	Full-band	89.94 ± 1.53	90.93 ± 1.80	89.15 ± 1.76	95.53 ± 0.87
	Low-band	79.24 ± 1.69	78.52 ± 1.56	80.37 ± 1.77	85.85 ± 0.89
	High-band	86.59 ± 1.27	87.18 ± 1.41	85.42 ± 1.39	93.31 ± 0.55
	MBNF	91.96 ± 1.75*	94.35 ± 1.65*	**90.41 ± 1.33**	98.02 ± 0.33*
eMCI vs. lMCI	Full-band	80.96 ± 1.34	79.51 ± 2.07	82.26 ± 2.14	89.52 ± 0.31
	Low-band	67.09 ± 1.35	64.80 ± 2.03	69.52 ± 1.57	75.09 ± 0.64
	High-band	79.35 ± 1.52	77.64 ± 1.59	82.15 ± 0.98	88.23 ± 0.57
	MBNF	90.96 ± 1.43*	92.26 ± 1.35*	90.81 ± 1.98*	97.65 ± 0.49*
AD vs. NC	Full-band	89.34 ± 1.30	84.04 ± 1.32	93.62 ± 1.75	96.45 ± 0.39
	Low-band	86.17 ± 1.80	81.13 ± 1.67	88.66 ± 0.78	90.17 ± 0.48
	High-band	86.96 ± 1.31	80.33 ± 1.25	92.78 ± 1.82	95.83 ± 0.53
	MBNF	**92.86 ± 1.69**	88.82 ± 1.44*	95.98 ± 1.55*	**97.77 ± 0.58**

*Denotes that the result of MBNF is significantly better than other competing schemes. Bold values indicate the best results in each task.

(1) The proposed scheme with multi-band fusion networks is significantly superior to other three competing schemes. This indicates that combining the various information of multi-band FBNs helps boost the classification performance for brain disease classification.
(2) The low-band scheme achieves a worse performance when compared with the high-band scheme in every classification task. Combined with previous researches (22, 23), the possible reason is that the features of high band-based FBNs are more robust and discriminative. For example, Zuo et al. have shown that the test–retest reliability of high-band-based fluctuations is greater and more widely distributed than that of the low-band (24).
(3) Regarding four tasks of classification based on two FBN construction methods, the task of identifying subjects with AD from normal controls is relatively easier. The underlying reason is that brain function degeneration in AD subjects could be more serious than MCI and NC.

### 3.4 Discriminative functional connections and brain regions

**FIGURE 5:**
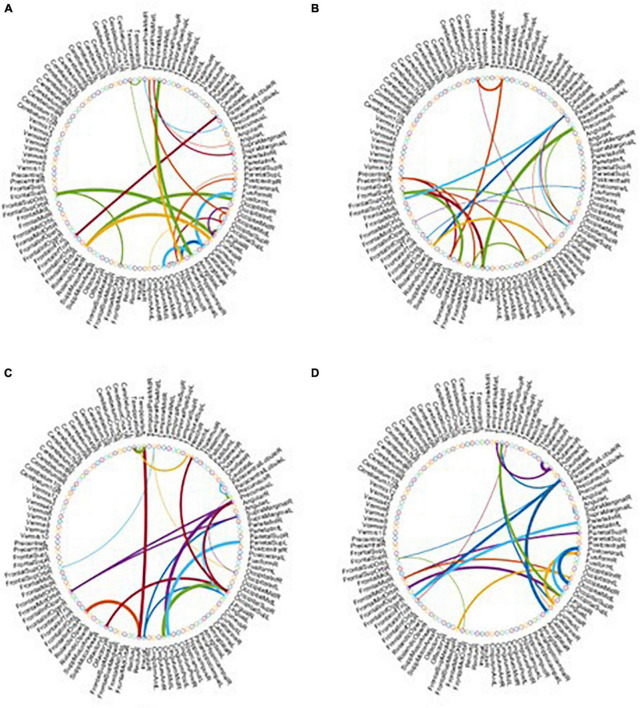
Most discriminative functional connections in four classification tasks: **(A)** eMCI vs. NC, **(B)** lMCI vs. NC, **(C)** AD vs. NC, and **(D)** eMCI vs. lMCI.

As the most important step in FBN analysis, selecting the discriminative features is meaningful to search for the biomarkers used to determine brain disease. A rising corpus of research indicates that many mental diseases emerge from interactions between various brain regions rather than being restricted to just one particular area of the brain. Therefore, we employ *t*-test to select the most discriminative functional connections for our MBFN method in four tasks of classification. As shown in Figure 5, the color of each arc is chosen at random for better visualization, and its thickness represents the discriminative power of connection (rather than the actual connectivity strength).

**FIGURE 6:**
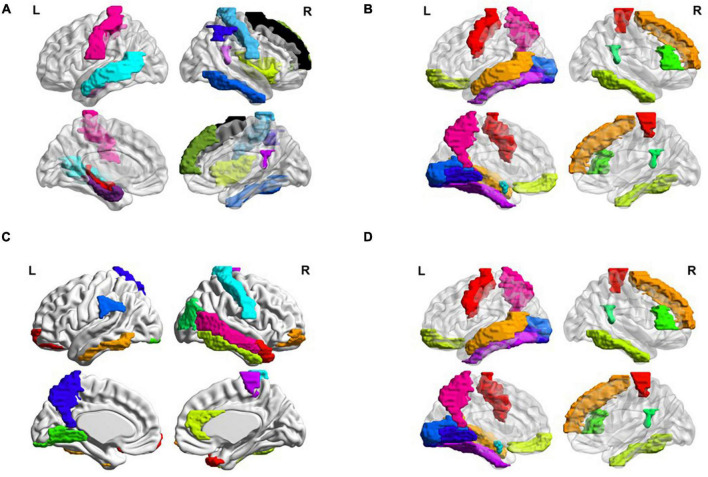
Most discriminative regions of interests (ROIs) identified by our proposed MBFN method in four tasks of **(A)** eMCI vs. NC, **(B)** lMCI vs. NC, **(C)** AD vs. NC, and **(D)** eMCI vs. lMCI.

Besides, we also visualized the discriminative brain regions based on the functional connections in Figure 6. This visualization is drawn by BrainNet Viewer toolbox^1^ and these stably selected brain regions are mapped onto the International Consortium for Brain Mapping (ICBM) 152 surface based on AAL atlas. For MCI classification (eMCI vs. NC and lMCI vs. NC), we can observe that frontal lobe, Cingulum, Postcentral, Fusiform and inferior temporal gyrus are the most discriminative brain regions. Previous research has shown that abnormal changes in these brain regions accelerates the conversion of people with mild cognitive impairment to Alzheimer’s disease (25–30). Similarly, for AD classification, the regions of the posterior cingulate gyrus, postcentral gyrus, c, hippocampus, middle temporal gyrus, and inferior temporal gyrus are the most discriminative brain areas, which have been previously documented to be involved in AD (31–34).

Many brain disorders are not isolated to specific brain regions, but result from the interaction of different brain regions. For example, the frontal lobe plays a key role in non-task long-term memory (35), the hippocampus is responsible for storage and transformation of long-term memory and spatial memory and localization (36), and the posterior cingulate gyrus is involved in processes such as emotion and self-evaluation (37). Memory loss, cognitive decline and frequent mood swings are hallmarks of Alzheimer’s disease (38). Previous studies have shown differences in the connections between these brain regions between AD patients and normal controls. These characteristics could be considered as biomarkers of Alzheimer’s disease (39).

### 3.5 Frequency variability of brain regions

**FIGURE 7:**
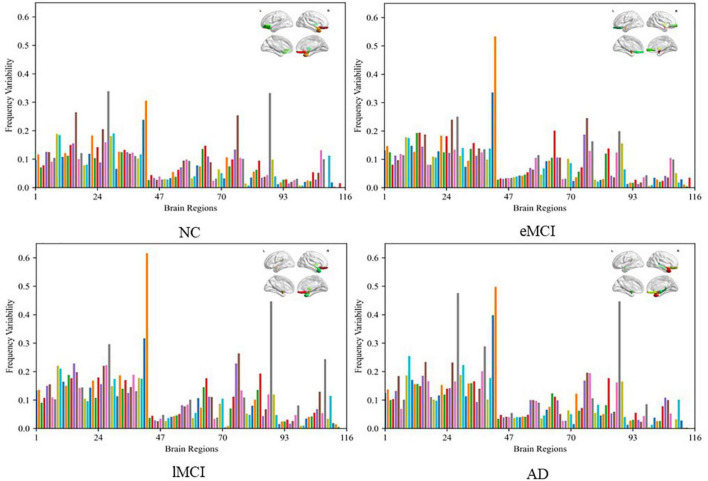
Frequency variability of all regions of interests (ROIs) in four categories (i.e., NC, eMCI, lMCI, and AD). The top right in every figure represents the ROI with the highest frequency variability (FV).

To visually illustrate the difference between high-and low-frequency BOLD signals, we employ frequency variability (FV) to assess changes in different brain regions at different frequency bands (40). FV is defined as follows:


(11) $$F\,V_{i}=1-\frac{\sum_{f=1,g\neq f}^{N_{F}}c\,o\,r\,r\,c\,o\,e\,f\,\left(F\,C_{f,i},F\,C_{g,i}\right)}{N_{F}\times \frac{N_{F}-1}{2}}$$

where *FC*_*f*,*i*_ is the functional connection of node *i*(*i* = 1,⋯,116) to other ROIs in frequency band *f*, *N*_*F*_ is the total number of frequency bands (here *N*_*F*_ = 2). The higher the value of FV, the greater the difference of brain regions in different frequency bands. Figure 7 shows the FV of all ROIs in four categories (i.e., NC, eMCI, lMCI, and AD).

We can observe that the FV of normal people is relatively lower than other patient categories. The probable reason is that the disease of MCI/AD can cause disturbance of normal neuronal behavior and destruction of neuronal networks, which leads to unstable BOLD signals. In addition, for patient categories (i.e., eMCI, lMCI, and AD), the amygdala, middle temporal gyrus, and superior frontal gyrus showed relatively high FV, which may be biologically associated with MCI/AD.

## 4 Discussion

In this section, we first analyze the effect of different signal decomposition methods, the impact of different fusion methods on classification performance, the effect of Different Datasets, and the effect of Connection Variations in FBNs. Then we present the limitations of this work as well as several future research directions.

### 4.1 Effect of different signal decomposition methods

In our proposed MBNF scheme, the EEMD signal decomposition method is used to extract different frequency band signals. To verify the effectiveness of the EEMD method and the effect of different signal decomposition methods on our experiment, we employ three different signal decomposition competing methods, including (1) discrete wavelet transform (DWT) (41), (2) local mean decomposition (LMD) (42), and (3) empirical mode decomposition (EMD). For a fair comparison, all competing schemes are performed in consistent steps (i.e., same data pre-processing, FBN construction and fusion, feature selection, and classification) except for the signal decomposition step.

Table 4 summarizes the results of four signal decomposition methods in two classification tasks. We can observe that our proposed MBNF using the EEMD decomposition method provides the best results. The probable reason is that EEMD can decompose signals adaptively according to the time characteristics of data, which has the advantage of obtaining good results in processing BOLD signals.

**TABLE 4:** Classification results of four signal decomposition methods in two tasks.

Task	Method	ACC (%)	SEN (%)	SPE (%)	AUC (%)
AD vs. NC	DWT	88.10	78.77	95.13	95.77
	LMD	86.15	73.13	95.71	93.26
	EMD	88.13	83.74	92.67	97.20
	Ours	**93.08**	**86.96**	**96.73**	**98.58**
eMCI vs. lMCI	DWT	85.80	84.19	87.86	93.96
	LMD	85.80	**87.89**	85.87	92.11
	EMD	87.74	85.49	90.76	96.21
	Ours	**90.64**	86.02	**94.97**	**96.98**

Bold values indicate the best results in each task.

### 4.2 Effect of different fusion methods

We use the SNF method to combine FBNs based on different frequency bands in the proposed MBNF scheme. To verify the effectiveness of the SNF method and the effect of different network fusion methods in our experiment, two methods are used to compare the SNF method, including (1) Concatenate, a scheme for splicing FBNs based on different bands into a feature vector; (2) Canonical Correlation Analysis (CCA), a typical fusion method (43). For a fair comparison, all competing schemes are performed in consistent steps (i.e., same data pre-processing, signal decomposition, FBN construction, feature selection, and classification) except for the FBN fusion step.

**FIGURE 8:**
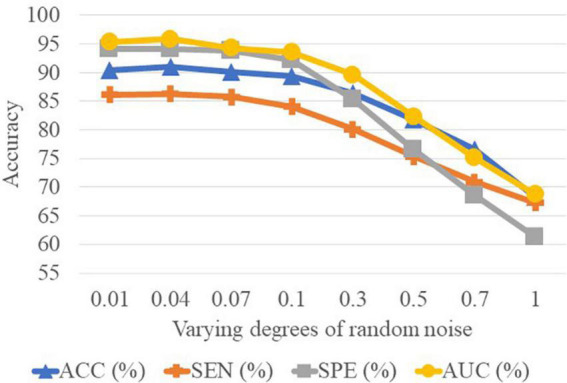
Results achieved by the proposed method with varying degrees of functional brain network (FBN) random noise of Pearson’s correlation (PC) in lMCI vs. eMCI classification.

In Table 5, we can observe that the performance of CCA is worse than the SNF techniques. The underlying reason is that CCA can only determine the linear correlation and ignore the nonlinear correlation in the interaction between the high-frequency FBN and the low-frequency FBN. Besides, the reason why the SNF achieves better performance than concatenating is that the concatenate method ignores the structural properties of FBNs by the splicing technique. To explore the impact of noise on FBN, we added random white Gaussian noise with varying standard deviation to the FBN (44). It can be seen in the Figure 7 and table that with the increasing noise level, the classification accuracy was decreasing. We used a bootstrapping method to enhance the robustness of our method. We resampled the data and created several training sets which were the same size as the original data. The experimental results are shown in the Figure 8.

**TABLE 5:** Classification results of three network fusion methods in two tasks.

Task	Method	ACC (%)	SEN (%)	SPE (%)	AUC (%)
AD vs. NC	Concatenate	90.88	84.04	96.02	97.40
	CCA	85.36	75.03	92.97	90.79
	Ours	**93.08**	**86.96**	**96.73**	**98.58**
eMCI vs. lMCI	Concatenate	87.74	**88.52**	87.53	94.43
	CCA	78.38	67.66	88.23	85.15
	Ours	**90.64**	86.02	**94.97**	**96.98**

Bold values indicate the best results in each task.

### 4.3 Effect of different datasets

Since different distributed datasets may affect the experimental results, we perform three independent datasets to confirm our conclusions, including Schizophrenia (SZ), Major Depressive Disorder (MDD), and Autism Spectrum Disorder (ASD). Specifically, the dataset of SZ (45), from publicly shared online datasets by the Mind Research Network and the University of New Mexico, includes 57 patients with chronic schizophrenia patients and 64 NCs. Besides, we also perform our proposed scheme on the ABIDE database (46) collected from the New York University site. The ABIDE dataset includes 184 subjects, of which 79 are from ASD and 105 are from NC. The MDD dataset is from the ninth site of the REST-meta-MDD Consortium (47), which contains 49 MDD patients and 47 NCs. Note that due to the fact that the MDD database used in this study is provided as preprocessed by the REST meta-MDD project, we have no control over the preprocessing pipeline. Therefore, we process the other two databases *via* the same pipeline as the MDD database for fairness.

As shown in Table 6, our MBNF method achieves the overall best performance regardless of which database is used. These results imply that combining the structural information of functional brain networks in different frequency bands helps to improve the accuracy of identifying patients from NCs. In addition, the other three databases give lower performance compared to the ADNI database. The probable reason is that the lesions of brain structure caused by AD/MCI are more severe than mental disease (e.g., MDD and ASD) and neurodevelopmental disorders (e.g., SZ).

**TABLE 6:** Classification result of three data sets on MBNF method.

Task	Scheme	ACC (%)	SEN (%)	SPE (%)	AUC (%)
Schizophrenia vs. NC	Full-band	60.52	58.37	65.83	68.57
	Low-band	56.74	55.56	63.15	65.55
	High-band	55.81	52.19	60.23	63.08
	MBNF	**61.34**	**59.89**	**66.71**	**69.83**
ASD vs. NC	Full-band	64.86	60.24	69.28	71.86
	Low-band	57.21	55.18	58.36	60.86
	High-band	60.73	56.83	62.61	63.73
	MBNF	**65.58**	**61.36**	**69.72**	**72.59**
MDD vs. NC	Full-band	59.67	61.64	58.73	62.27
	Low-band	54.46	54.28	53.61	57.95
	High-band	56.69	56.39	54.41	59.34
	MBNF	**60.93**	**59.36**	**60.79**	**63.82**

Bold values indicate the best results in each task.

### 4.4 Effect of connection variations in FBNs

It is well-known that PC based functional connectivity tends to be sensitive to noise. To investigate whether variations in connectivity affect our proposed method, we performed a set of experiments by adding white Gaussian random noise of varying degrees to the FBN estimated by the PC, and present the experimental results in Figure 8. It can be observed that the classification results only show a slight fluctuation when the noise degree (standard deviation) is less than 0.1. However, the classification accuracy decreases substantially as the noise level increases. This side-fact indicates that low degrees of noise have little effect on our method and implies that the MBNF scheme already has a relatively good robustness.

### 4.5 Limitation and future work

Although our proposed framework has a good effect on disease diagnosis, there are still several limitations that need to be noted. The steps of signal decomposition, FBN construction, and fusion in our proposed MBNF scheme are performed separately, which probably leads to potential noise in each step. In addition, the extracted features based on the way of separate-step are not necessarily optimal for the subsequent classification task. Therefore, an end-to-end method like deep learning improves experimental performance, which is also the direction of our future work.

## 5 Conclusion

In this paper, we propose a multi-frequency network Fusion framework (MBNF) to combine the structural information of functional brain networks in different frequency bands. Specifically, we first use EEMD to decompose the BOLD signal into high-frequency signal and low-frequency signal. Then we construct a high-frequency functional network and a low-frequency functional network, respectively. Finally, the similarity network fusion is employed to fuse high-frequency network and low-frequency network for classification. The validation on the ADNI dataset shows that our proposed multi-band network fusion framework is effective.

## Data availability statement

The original contributions presented in this study are included in the article/supplementary material, further inquiries can be directed to the corresponding author.

## Author contributions

LG and ZW designed the study. LG and YZ downloaded and analyzed the data, performed experiments, and drafted the manuscript. QL and KG preprocessed the data and performed some experiments. YZ and ZW revised the manuscript. All authors read and approved the final manuscript.

## Footnotes

1
https://www.nitrc.org
